# Melioidosis in Singapore: Clinical, Veterinary, and Environmental Perspectives

**DOI:** 10.3390/tropicalmed3010031

**Published:** 2018-03-12

**Authors:** Siew Hoon Sim, Catherine Ee Ling Ong, Yunn Hwen Gan, Dongling Wang, Victor Wee Hong Koh, Yian Kim Tan, Michelle Su Yen Wong, Janet Seok Wei Chew, Sian Foong Ling, Brian Zi Yan Tan, Agnes Zhengyu Ye, Patrick Chuan Kiat Bay, Wai Kwan Wong, Charlene Judith Fernandez, Shangzhe Xie, Praveena Jayarajah, Tasha Tahar, Pei Yee Oh, Sonja Luz, Jaime Mei Fong Chien, Thuan Tong Tan, Louis Yi Ann Chai, Dale Fisher, Yichun Liu, Jimmy Jin Phang Loh, Gladys Gek Yen Tan

**Affiliations:** 1 Defence Medical and Environmental Research Institute, DSO National Laboratories, Singapore 117510, Singapore; ssiewhoo@dso.org.sg (S.H.S.); catong@dso.org.sg (C.E.L.O.); dongling@dso.org.sg (D.W.); kweehong@dso.org.sg (V.W.H.K.); tyiankim@dso.org.sg (Y.K.T.); wsuyen@dso.org.sg (M.S.Y.W.); cseokwei@dso.org.sg (J.S.W.C.); lsianfoo@dso.org.sg (S.F.L.); lyichun@dso.org.sg (Y.L.); jimmyloh@dso.org.sg (J.J.P.L.); 2 Department of Biochemistry, Yong Loo Lin School of Medicine, National University of Singapore, Singapore 117597, Singapore; bchganyh@nus.edu.sg; 3 Laboratories Group, Agri-Food & Veterinary Authority of Singapore, Singapore 718827, Singapore; Brian_TAN@ava.gov.sg (B.Z.Y.T.); agnes_ye@ava.gov.sg (A.Z.Y.); Wong_Wai_Kwan@ava.gov.sg (W.K.W.); Charlene_FERNANDEZ@ava.gov.sg (C.J.F.); 4 Food Establishment Regulation Group, Agri-Food & Veterinary Authority of Singapore, Singapore 608550, Singapore; chuan_kiat@ava.gov.sg; 5 Conservation, Research and Veterinary Services, Wildlife Reserves Singapore, Singapore 729826, Singapore; shangzhe.xie@wrs.com.sg (S.X.); praveenajayarajah@yahoo.com (P.J.); tasha.tahar@wrs.com.sg (T.T.); peiyee.oh@wrs.com.sg (P.Y.O.); sonja.luz@wrs.com.sg (S.L.); 6 Department of Infectious Diseases, Singapore General Hospital, Singapore 169608, Singapore; Jaime.chien.m.f@singhealth.com.sg (J.M.F.C.); tan.thuan.tong@sgh.com.sg (T.T.T.); 7 Division of Infectious Diseases, Department of Medicine, National University Health System, Singapore 119228, Singapore; louis_chai@nuhs.edu.sg (L.Y.A.C.); mdcfda@nus.edu.sg (D.F.); 8 Department of Medicine, Yong Loo Lin School of Medicine, National University of Singapore, Singapore 117597, Singapore; 9 National University Cancer Institute, Singapore 119074, Singapore

**Keywords:** melioidosis, *B. pseudomallei*, Singapore, clinical, veterinary, environmental

## Abstract

Melioidosis is a notifiable infectious disease registered with the Ministry of Health (MOH) and Agri-Food & Veterinary Authority (AVA), Singapore. From a clinical perspective, increased awareness of the disease has led to early detection and treatment initiation, thus resulting in decreasing mortality rates in recent years. However, the disease still poses a threat to local pet, zoo and farm animals, where early diagnosis is a challenge. The lack of routine environmental surveillance studies also makes prevention of the disease in animals difficult. To date, there have been no reports that provide a complete picture of how the disease impacts the local human and animal populations in Singapore. Information on the distribution of *Burkholderia pseudomallei* in the environment is also lacking. The aim of this review is to provide a comprehensive overview of both published and unpublished clinical, veterinary and environmental studies on melioidosis in Singapore to achieve better awareness and management of the disease.

## 1. Introduction

Melioidosis is an infectious disease associated with high mortality and morbidity in endemic regions of Southeast Asia and northern Australia [1]. The etiological agent, *Burkholderia pseudomallei*, can be found in the soil and water in these endemic regions. Infection generally occurs through inhalation, ingestion or contact of skin wounds with contaminated soil, dust particles or water [2]. Melioidosis, also known as the great mimicker, can result in highly diverse disease manifestations, often complicating diagnosis and delaying treatment [3,4,5,6]. Other than humans, *B. pseudomallei* can also infect a wide range of animals including goats, sheep, camels, birds, crocodiles, kangaroos, etc. [7,8]. The zoonotic potential of *B. pseudomallei* (Bp) has also been reported [7,9]. Due to the hardy nature of the bacterium, it is able to survive in the most nutrient-limiting environments, such as distilled water [10,11].

The first case of melioidosis in Singapore was reported in 1920 [12]. Since the 1980s, rapid urbanization has occurred within the country, and today it is mainly dominated by high-rise buildings and well-developed infrastructure. However, despite its urbanized setting, melioidosis still exists in Singapore, with an overall annual incidence of 0.6–2.4 per 100,000 of the population between the years 2000 and 2015, as reported by MOH [13]. Furthermore, animal cases of melioidosis were reported as early as in the 1980s by AVA (formerly called Primary Production Department). In this review article, we describe the clinical, veterinary and environmental aspects of melioidosis in Singapore.

## 2. Clinical Aspects

### 2.1. Disease Epidemiology

Prior to 1989, there were only two published reports on the epidemiology of the disease in the country. Tan et al. (1990) [14] reported a sudden increase in the number of cases from 5 cases in 1987 to 36 and 24 cases in the years 1988 and 1989, respectively, and attributed the increase to the generation of infective aerosols as a result of soil excavation activities conducted around the island. The disease was also a concern among healthy military personnel due to involvement with soil-related training programs during these early years. Between 1987 and 1994, 23 cases (including 4 fatal cases) were reported [15].

**Figure 1:**
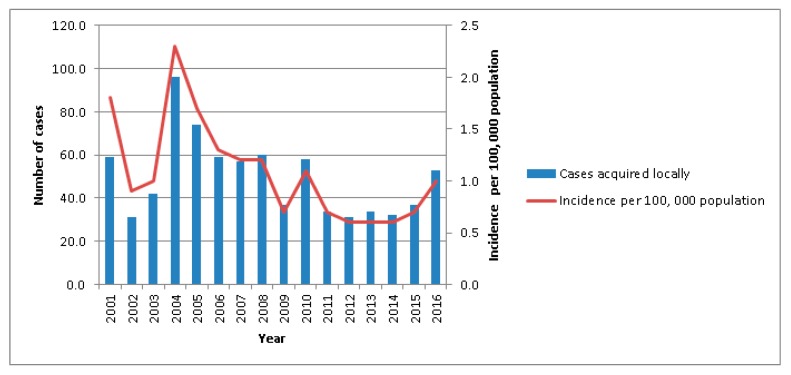
Annual number of human melioidosis cases from 2003 to 2016 in Singapore.

Three separate studies addressing the epidemiology of melioidosis were conducted, from 1989 to 1996, 1998 to 2007, and 2003 to 2014 [16,17,18]. The mean annual number of melioidosis cases reported during these three study periods were 46, 70, and 44, respectively. The higher mean annual number observed during the period from 1998 to 2007 was related to a melioidosis outbreak in 2004, which was associated with higher-than-average rainfall [17]. A molecular typing study confirmed that this 2004 outbreak was caused by highly heterogeneous *B. pseudomallei* isolates, rather than a single virulent strain [19]. In the last-mentioned epidemiological study, spanning 2003–2014, a progressive 10% reduction in the incidence of melioidosis cases was noted. However, the last 2 years (2015–2016) have seen a rise in the number of melioidosis cases diagnosed (Figure 1). The early case-fatality rate, as exemplified by the 98 cases that occurred during the 2004 surge, was 52.6% [17]. The mortality rate has since fallen to 18.4%, possibly as a result of increased disease awareness and earlier recognition by clinicians, and prompt institution of definitive antimicrobial treatment.

In Singapore, the recent overall annual melioidosis incidence rate was 1.1 per 100,000 population. The incidence rate was higher in those of Malay and Indian ethnicity (2.4 and 2.1 per 100,000 respectively) [18]. Males were predominantly affected, which can possibly be attributed to their involvement in outdoor occupational or recreational activities [16,17,18]. Systemic melioidosis primarily affects older individuals with underlying medical conditions, with the highest disease incidence rate being reported in the population cohort ≥45 years of age. Diabetes mellitus is the key co-morbidity, reported in 47.9% to 56.7% of the diagnosed cases [16,17,18]. Patients with renal impairment were also at higher risk of infection.

### 2.2. Clinical Features of Melioidosis Patients

In Singapore, the major systemic presentations of melioidosis are bacteraemia (60.3%), deep organ abscesses (40.7%) and pneumonia (33.1%) [18]. In contrast to *B. pseudomallei* bacteraemia cases, the incidence of cases presenting as pneumonia is on the decline. Conventionally, antimicrobial regimens containing *B. pseudomallei*-active agents (e.g., ceftazidime or a carbapenem) have been utilized for treatment of severe community-acquired pneumonia in Singaporean intensive care units. For deep abscesses, the primary organs involved are liver, prostate and spleen, and these are often seen in patients with poorly-controlled diabetes. The incidence of deep organ abscesses appears to be on the rise [18,20,21]. Localized soft tissue infections have been seen, and some of these cases may be linked to local trauma or inoculation. These patients often do not have diabetes or compromised immunity, in contrast to the patients with bacteraemia or deep abscesses.

Singapore has also seen its share of melioidosis cases with uncommon clinical presentations. For example, in 2006, there was a case of melioidosis osteomyelitis reported in a 32-year-old diabetic man, who also had infection in the spleen and liver [22]. In another case, a 37-year-old Italian man possibly acquired melioidosis through inhalation of dust generated by helicopter rotor blades during business travel to Singapore. He developed bacteraemia, pneumonia, splenic abscess and osteomyelitis of the head of humerus [23].

Involvement of the cardiovascular system, a rare occurrence associated with high morbidity and relapse rates, has also been observed in melioidosis cases in Singapore [24]. Rao et al. (2009) [25] reported two cases presenting with abdominal aortic pseudo-aneurysms requiring both medical and surgical intervention, with only one surviving. A relapsing case of meningitis caused by *B. pseudomallei* following inadequate treatment of the primary episode of melioidosis mycotic aneurysm was also reported by Chlebicki et al. (2008) [26]. Between 1997 and 2000, five cases presenting with brain abscess were also reported [27]. Successful treatment was achieved with drainage coupled with antibiotics, although two of the cases failed to achieve complete neurological recovery [28].

### 2.3. Diagnosis

In Singapore, the reference standard for the diagnosis of melioidosis is based on the culture of *B. pseudomallei* from clinical specimens [3,29], along with relevant clinical presentations. Biochemical tests including API 20NE (bioMerieux, Lyon, France) and Vitek 2 GN card (bioMerieux, Lyon, France) are also conducted to confirm the biochemical properties of the isolated *B. pseudomallei*. Since the occurrence of 4 deaths in 23 melioidosis cases among apparently young and healthy military personnel between 1987 and 1994, melioidosis has been a priority consideration for Singapore’s clinicians [15,30]. Risk factors including activities entailing soil and water contact, as well as underlying co-morbidities such as diabetes and renal disease, will also raise diagnostic concerns among the clinicians [31].

A disadvantage of culture-based diagnosis is the delay of some days. Molecular methods such as Bp-specific polymerase chain reactions (PCR) targeting bacterial DNA fragments provide rapid diagnosis for melioidosis, but are not routinely used in local clinical laboratories and the sensitivity of PCR can be low, especially with blood specimens [32,33]. While a serological test alone cannot offer a definitive diagnosis of melioidosis, especially in endemic areas [34], seropositivity is an indicator of exposure to *B. pseudomallei*. The most commonly used serological test for a melioidosis diagnosis is indirect hemagglutination assay (IHA), which has been well evaluated and accepted since it is cost-effective and has a short turnaround time, although in other countries it has been reported to lack both sensitivity and specificity [3].

In Singapore, the IHA with a cut-off ratio of ≥1:16 for seropositivity was first established by Yap et al. (1991) [35]. The DSO Clinical Diagnostic Services Laboratory (CDSL) has been ISO15189 accredited for performing the IHA test to facilitate melioidosis diagnosis for local samples. The laboratory has conducted IRB-approved studies (Singhealth Centralized Institutional Review Board, DSO IRB committee) and performed IHA tests on serum samples collected from culture-confirmed melioidosis cases, clinically unconfirmed cases, and healthy volunteers from 2004 to 2016. Based on unpublished data (Table 1), it has been observed that all of the culture-confirmed melioidosis cases were also positive by IHA, 54.8% with very high (≥1:512) and 38.7% with high (1:128 to 1:256) titers. In contrast, the two large cohorts (992 and 1027 cases) with suspected melioidosis or clinically unconfirmed infection were mostly negative in the IHA test (89.4% and 86.5%, respectively). Furthermore, of 109 healthy volunteers, 23 (21.1%) had low positive results (1:16 to 1:64) and one subject (0.9%) was highly positive with an IHA titer of 1:128, suggesting previous exposure to *B. pseudomallei*, possibly due to engagement in prior outdoor activities. The IHA is thus considered more useful in Singapore than elsewhere, and those with high or very high IHA titers are considered likely either to be melioidosis cases with active Bp infection or individuals who have had prior exposure to *B. pseudomallei*. 

### 2.4. Treatment

Clinicians in Singapore, particularly physicians, intensivists and emergency department specialists, have melioidosis in mind early when a suspicious clinical pattern presents. Empiric antibiotics are generally started pending culture results. Hence, ceftazidime or a carbapenem are featured in local empiric antibiotic guidelines for severe community-acquired pneumonia for this reason. The treatment for melioidosis in Singapore is conducted in accordance with international guidelines, consisting of an initial intensive phase followed by an extended eradication phase [37]. As initial empiric therapy for suspected melioidosis or for intensive phase treatment for confirmed melioidosis, either ceftazidime or meropenem is administered intravenously. The duration of treatment for the intensive phase is at least 2 weeks. Some clinicians may extend treatment duration to 4 weeks or beyond, in circumstances such as multiple large deep organ abscesses or extensive multi-lobar pneumonia. In addition, while not in the standard guidelines, some physicians also add oral trimethoprim-sulfamethoxazole during the intensive phase for the perceived benefit of good penetration into deep organs and lungs as well as the anecdotal emergence of ceftazidime resistance, despite the fact that such a practice has not been proven to be more efficacious [38]. The eradication phase is long, lasting at least 3 months, and consists of oral trimethoprim-sulfamethoxazole as the backbone of therapy. Locally, some physicians may opt to add in a second agent, either amoxicillin-clavulanate or doxycycline, in addition to trimethoprim-sulfamethoxazole. Drug tolerance is always a concern during the extended treatment periods, and so close follow-up is advised. In addition, due to the high prevalence of glucose-6-phosphate dehydrogenase (G6PD)-deficiency in this region, G6PD screening is advised before commencement of trimethoprim-sulfamethoxazole. For patients who are intolerant or who have known sulfonamide allergy, the combination of amoxicillin-clavulanate plus doxycycline is used, although this regimen is perceived to be less efficacious than one containing trimethoprim-sulfamethoxazole. Eradication therapy is considered adequate and ceases when follow-up clinical, radiological and biochemical (e.g., inflammatory markers such as C-reactive protein) assessments document complete resolution. Otherwise, the eradication phase treatment may be extended beyond 3 months. In some circumstances (e.g., disease complexity or drug allergy), treatment may deviate from the standard antibiotic regimens [31]. In Singapore, antibiotic susceptibility testing is routinely performed on *B. pseudomallei*, with alternative antibiotics used when resistance to the standard antibiotics is detected. In 2010, Kung et al. [39] reported a patient with mediastinal lymphadenitis, who developed resistance to ceftazidime during the course of treatment. One of us has managed another patient with melioidosis, whose isolate developed ceftazidime resistance on treatment, in 2008 [40]. The patient relapsed with overwhelming bacteraemia and rapidly demised, prompting some clinicians to favor initial antibiotic therapy with a combination of two active antibiotics.

The management of deep organ abscesses is challenging. There is a need to ensure adequate drug penetration into the abscess cavity for adequate treatment, often necessitating an extended duration of treatment. To reduce the microbial burden, some local clinicians advocate radiologically-guided drainage of abscesses, where possible, especially in the liver and prostate. The rationale for such an invasive procedure is multi-fold: (i) for diagnostic microbiological confirmation of the disease, (ii) for *B. pseudomallei* susceptibility testing, (iii) to reduce the microbial burden, and (iv) to shorten treatment duration.

For severe septic melioidosis cases, in addition to antibiotic therapy, treatment in an intensive care unit (ICU) with strict glycemic control [41] is also employed to improve the outcome [31]. Careful use of anti-diabetic therapy in diabetic patients with septic melioidosis is recommended, and a recent study by Liu et al. (2014) [42] highlighted the occurrence of more severe complications in diabetic patients receiving sulphonylurea due to the suppression of the host inflammatory response by the drug.

## 3. Veterinary Aspects

### 3.1. Prevalence and Surveillance of Animal Melioidosis 

Singapore has a diverse population of animals. For livestock animals, there are a total of 5 poultry layer farms, 4 dairy (3 cattle and 1 goat) farms, 220 fish farms (food and ornamental) and 11 horse establishments (racing and equestrian) [43]. As of 2016, the pet population, comprising dogs, cats, birds, fish, and small mammals, is estimated to be 780,100 [44]. In addition, there is a zoological collection of 16,134 animals, consisting of 949 different species of Pisces, Amphibia, Reptilia, Aves and Mammalia in Wildlife Reserves Singapore (WRS).

In Singapore, animal melioidosis cases are notifiable to AVA. To date, AVA has documented 454 confirmed melioidosis cases between 1983 and 2016, comprising isolates from wild animals (including the zoo animals at WRS), pet animals from local veterinary clinics, farm animals, and the environment, such as outdoor multipurpose fields and ponds. At WRS, 37 sporadic cases in 26 animal species were documented between 1983 and 2017 (Table 2). Notably, gorillas appeared to be highly susceptible to melioidosis. In 1984, a total of 4 imported gorillas (2 from Monaco and 2 from Bristol zoo, imported between 1982 and 1983) succumbed to melioidosis. Additionally, of another 2 gorillas imported from Dublin zoo in 1992, one succumbed to melioidosis six months after importation. The other was subsequently returned to Dublin zoo in 1993. *B. pseudomallei* was cultured from various organs of these five deceased gorillas (Table 2). It was postulated that various factors, including social dynamics, diet and housing, had resulted in increased susceptibility of these gorillas to *B. pseudomallei* infection.

**Figure 2:**
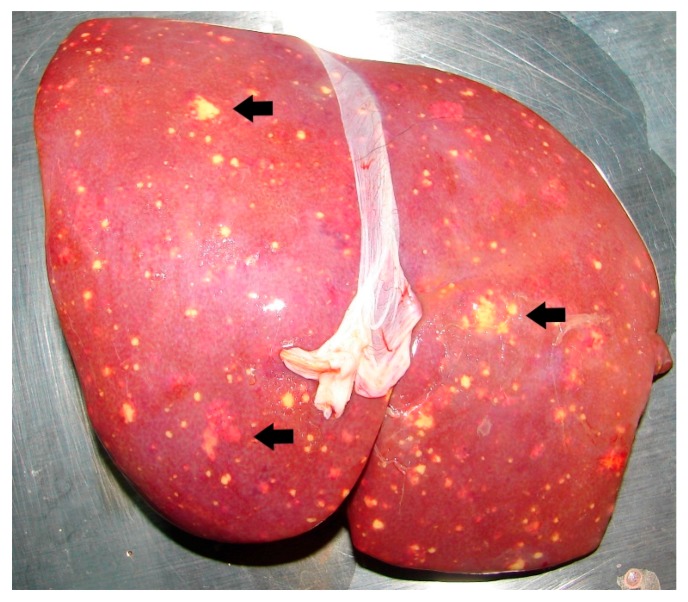
Multifocal abscess (black arrows) in the liver of a Douc langur with melioidosis.

**Figure 3:**
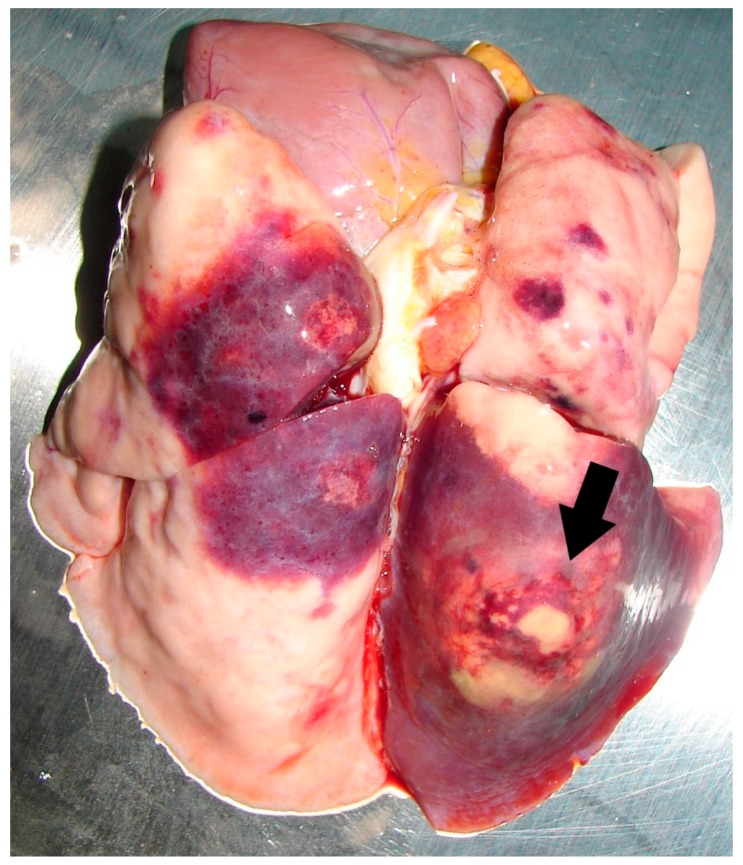
Large abscess in the left caudal lung lobe (black arrow) of a Douc langur with melioidosis.

Clinical signs can differ within each animal species, depending on the site of infection [45]. Based on past observations, the first clinical signs in infected animals were non-specific and consisted of inappetence, lethargy and diarrhea. In some cases, infected animals can even succumb to infection within one or two days without showing visible signs of disease.As previously reported, one key feature of animal melioidosis was the formation of abscesses in one or multiple organs [7,46]. At WRS, the most common sites of lesions in infected animals were the liver (Figure 2) and lungs (Figure 3). Lung lesions were more commonly seen in primates [47], whereas liver lesions were more common among the avian cases (78%). It is also interesting to note that lung lesions have never been seen in the avian cases.

With the increase in global trade of animals for food, zoological exhibitions and as pets, importation and movement of subclinically-infected animals poses a significant risk to local animal populations due to the propensity of *B. pseudomallei* for long latency periods. These animals can shed *B. pseudomallei* in their faeces or through ruptured abscesses during the stressful transport process and change in environment, which might serve as a source of infection or environmental contamination when they are in close contact with naïve animals [48,49].

There is no formal active surveillance program for melioidosis in local pet, zoo or farm animals. In Singapore, farm animals are mainly reared for dairy products and eggs, and are not slaughtered for meat. In addition, only pasteurized milk is allowed to be sold for consumption. To date, there has only been a single instance in which *B. pseudomallei* was isolated from the spleen of a clinically healthy dairy goat from a local farm in 2014. However, subsequent sampling of the goats in the farm did not yield any positive cases. As part of Singapore’s food safety program, AVA conducts post-mortem inspection on imported livestock, i.e., pigs and accompanying viscera (spleen, liver, kidneys) at the local pig abattoir. The inspection involves visual checks, palpation and, where necessary, incision to check for abnormalities, including abscesses. Carcasses and viscera with multiple abscesses/pyemia will not enter the food chain, as they will be condemned and subsequently incinerated if the examination findings are consistent between AVA inspectors and Supervising Inspectors (SI). In addition, AVA conducts regular microbiological surveillance on the condemned carcasses and viscera (liver, lymph node, lung, spleen and kidney), which includes *B. pseudomallei* culture, to monitor the health status of imported livestock. From January 2008 to December 2016, a total of 1696 samples were collected, of which 341 (20%) were positive for *B. pseudomallei*. Thus, the risk of transmission from infected animals to humans through the consumption of meat and animal products would be deemed negligible. 

Ongoing work is being done to analyze the strains isolated from animals. The local farms also have good animal husbandry practices in place and are required to report any abnormal clinical signs observed. Furthermore, the health status of farm animals is also inspected regularly by AVA. These additional measures provide some forms of surveillance for any first signs of disease in farm animals.

### 3.2. Laboratory Diagnosis for Animal Melioidosis

The diagnosis of melioidosis in animals requires the isolation and identification of *B. pseudomallei,* usually from swabs or abscesses from affected organs. Diagnosis of the reported animal cases is usually obtained through post-mortem examination, unless the presenting clinical sign is a lesion on the skin, as observed in a Chinese goral (*Naemorhedus griseus*) in 2008. Upon detection of the disease, environmental sampling from the surrounding soil and water where the animals reside is conducted as part of the investigation procedure for source attribution.

In all cases, the standard Ashdown media or modified Ashdown broth containing colistin and crystal violet [51] are used for the isolation of *B. pseudomallei*. Further confirmation is achieved through phenotypic characterization using biochemical tests, including the API 20NE kit or Vitek GN card, and genotypic characterization using *B. pseudomallei*-specific real-time PCR [52].

### 3.3. Treatment and Prevention of Melioidosis in Zoo Animals

In Singapore, animals suspected of having melioidosis are generally given supportive treatment, which may include fluid therapy, anti-inflammatories and antibiotics. In the four parks managed by WRS, as a preventive measure against melioidosis, proper water hygiene and environmental surveillance are conducted to identify for the presence of *B. pseudomallei* in the exhibit areas. Quarterly environmental sampling is conducted before introducing susceptible species into the parks. For instance, between February 2016–2017, a total of 450 soil samples and 54 water samples were collected from four sites, namely the Butterfly Park, Kidzworld, Chimpanzee Exhibit and Polar Bear Exhibit. All samples were negative for *B. pseudomallei* by both culture and real-time PCR. The Chimpanzee Exhibit was positive for *B. multivorans* in May 2016, but subsequent soil and water samples collected from the same site were negative for the bacterium. 

## 4. Environmental Melioidosis

### 4.1. Prevalence and Environmental Surveillance of B. pseudomallei

For a country long known to be endemic for melioidosis, Singapore has surprisingly few publications on the isolation, distribution, prevalence and spread of the pathogen in the local environment. After the first case of melioidosis in 1920 [12], the next report on environmental isolation of *B. pseudomallei* was in 1971 [53]. In this study, 8 of the 136 surface water samples collected within the city of Singapore were positive for *B. pseudomallei* (isolation rate of 5.9%) (Table 3). In 1995, Yap et al. [54] documented the recovery of 3 isolates from water samples collected from a moat within an animal enclosure in Singapore Zoological Gardens (now known as WRS), where the gorillas had earlier succumbed to melioidosis. Additionally, two soil isolates were obtained from an island off mainland Singapore where two fatal human cases of melioidosis had been reported, and one isolate from a residential compound where a pet German shepherd dog had died (Table 3) [54]. To date, the most extensive environmental surveillance of *B. pseudomallei* in Singapore involved the collection of 395 soil samples during the period 1992 to 1996 for epidemiological investigation of the disease [16]. Seven of the soil samples obtained from 3 different sites were positive for the pathogen (Table 3) [16].

Between 2000 and 2013, DSO National Laboratories executed a number of projects on environmental surveillance of *B. pseudomallei* (unpublished) (Table 4). One of these environmental surveillance studies was conducted in the island off mainland Singapore where Yap et al. (1995) [54] had previously reported the isolation of *B. pseudomallei*. Among the 188 soil samples collected from fields, plantations and reclaimed lands (*n* = 98, 62 and 28, respectively), and 16 water samples from streams or puddles, 3 soil samples from plantations (4.8%) and 2 water samples (12.5%) were positive for *B. pseudomallei*. In 2001, through collaborative efforts with WRS and Prof Paul Ananth Tambyah from the National University Hospital (NUH), 43 soil samples and 3 water samples were collected from 3 animal enclosures. No *B. pseudomallei* was isolated. 

Between 2003 and 2005, environmental surveillance of *B. pseudomallei* was carried out at 9 locations on mainland Singapore and 731 soil samples were collected. Two of these locations (Park C and Park D) tested positive for *B. pseudomallei*, with isolation rates of 1.0% and 3.3%, respectively (Table 4). Data from a subsequent surveillance project carried out from January to April 2013 showed that the isolation rate of *B. pseudomallei* from soil samples collected from a forested hill in a southern island off Singapore was 1.8%. However, rainwater and water samples collected from run-off from the forested hill were negative for *B. pseudomallei* (Table 4).

It is worth noting that environmental surveillance studies to identify the source of infection are confounded by the small area of Singapore, and the movement of the general population all over the island. In addition, technical and funding issues, and the difficulty in getting approval from regulatory bodies and private institutions have also hindered these environmental surveillance studies.

### 4.2. Detection of B. pseudomallei in Environmental Samples

Optimizing protocols for sample collection and laboratory processing for the isolation and identification of *B. pseudomallei* is one of the many technical challenges faced in environmental surveillance studies. Due to the diverse range of colony morphotypes of *B. pseudomallei* when cultured on Ashdown agar, considerable expertise is required for visual identification of the bacterial colonies [57]. The hamster inoculation method used by Thin et al. (1971) [53] is a long and tedious process, making it challenging to sample extensive areas and collect large numbers of samples. Similarly, Yap et al. (1995) [54] adopted a ‘small and sampling-biased’ approach in their study, thus limiting the extent of sampling area.

Following the dissemination of an internationally-recognized SOP for environmental sampling of *B. pseudomallei* by the Detection of Environmental *Burkholderia pseudomallei* Working Party (DEBWorP) in 2012, samples could be collected systematically and processed more efficiently [58]. Using this SOP, DSO embarked on an extensive island-wide environmental surveillance project for comprehensive understanding of the distribution, prevalence, spread, and characterization of environmental *B. pseudomallei* in the Singapore environment (manuscript in preparation). Moreover, with the recent success of the air sampling methodology reported by Currie et al. (2015) [59], we will be reassessing our air sampling protocols to increase our chances of isolating the pathogen from the air in Singapore.

### 4.3. Correlation between Environmental Isolates and Animal/Clinical Isolates

There have so far been few successes in directly correlating clinical isolates with environmental isolates in the Singapore context. Using restriction endonuclease (RE) analysis with pulsed-field gel electrophoresis (REA-PFGE), Henget al. (1989) [16] reported that the genotype of *B. pseudomallei* isolated from 4 soil samples in a locality was similar to that isolated from an elbow abscess of an adult who sustained an injury at the location. However, Yap et al. (1995) [54] reported that the genotypes of clinical isolates from 2 patients who died of melioidosis whilst staying in an island off Singapore did not match those recovered from the soil. Instead, the study had more successes with animal isolates—restriction endonuclease profiles of *B. pseudomallei* isolates obtained from infected gorillas in Singapore Zoological Gardens matched the isolates obtained from the surrounding moat of the animal enclosure. In addition, a soil isolate obtained from a residential compound matched the genotype of an isolate from a pet German shepherd dog that succumbed to the disease [54] (Table 3). It is likely that the relatively smaller and well-defined boundaries of the animal enclosure and residential compound increased the chances of locating the environmental source.

Advanced technologies, such as microarrays and multi-locus sequence typing (MLST) have allowed more detailed study of the clinical, animal and environmental *B. pseudomallei* isolates in Singapore. Sim et al. (2008 [47] performed high-resolution comparative genomic analysis to determine the relatedness of 94 *B. pseudomallei* isolates from Southeast Asian origins, and demonstrated a core genome and an accessory genome comprising of 86% and 14% of the genes (respectively) in the Bp K96243 genome. Of these isolates, 68 were isolated from clinical (*n* = 35), animal (*n* = 17) and environmental (*n* = 16) sources from Singapore. Bp 22, isolated from a fatal melioidosis case in a young and healthy individual in the 1980s, was amongst the clinical isolates studied. Seven environmental *B. pseudomallei* isolates, including Bp DB and Bp DC [54], Bp 12-40, Bp 15-10, Bp 15-40, Bp SW1 and Bp SW 9 isolated by DSO National Laboratories (herein referred as DSO isolates for easy reference), were obtained in the vicinity of the origin of Bp 22. Unsupervised clustering of the accessory genomes showed that Bp 22 and the DSO isolates belonged to a broad ‘Environmental’ cluster [47]. Interestingly, Bp DB and Bp DC did not cluster together with the ‘Environmental’ isolates, but belonged to a broad ‘Animal’ cluster, instead [47] (Table 5). The results achieved with MLST analysis of these isolates was in alignment with the genome clustering data, with Bp 22 and the DSO isolates belonging to ST423, and Bp DB and Bp DC belonging to ST51 (Table 5) [60].

**Figure 4:**
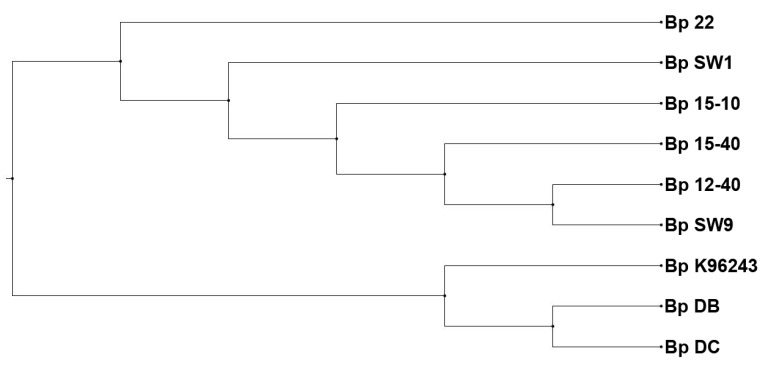
Whole genome phylogeny tree of clinical and environmental isolates from same locale in Singapore. Phylogenetic tree generated using Phylogenetic Tree Building Service by PATRIC v3.5.7 (https://www.patricbrc.org/app/PhylogeneticTree), depicting the relationships of the clinical isolate Bp22 and environmental isolates obtained from an island off Singapore. Bp K96243 was included as a reference isolate.

Furthermore, using whole genome sequencing (WGS), which provides a higher resolution of isolate matching, the seven environmental isolates were resolved into 2 clades: Clade A, which comprised Bp DB and Bp DC; and Clade B, which was made up of DSO isolates [60]. Unfortunately, the clinical isolate, Bp 22, was not included in this study. To examine the relatedness of Bp 22 with DSO isolates, the genome sequence of Bp 22 was obtained from a DSO in-house sequencing project and compared with the genome sequences of the seven environmental isolates. Using Phylogenetic Tree Building Service by PATRIC v3.5.7 (https://www.patricbrc.org/app/PhylogeneticTree), we determined that Bp 22 was indeed closely related to the 5 DSO isolates and was distinctly different from Bp DB and Bp DC (Figure 4).

### 4.4. Characterization of Environmental B. pseudomallei Isolates from Singapore

While much attention has been paid to characterization of clinical isolates, the environmental isolates from Singapore have been largely neglected in most studies. The antibiograms of clinical isolates from Singapore have been investigated in detail in various studies, reporting consistent susceptibility to ceftazidime, imipenem, amoxicillin-clavulanate, chloramphenicol and tetracycline [14,62,63]. Unfortunately, similar studies of environmental *B. pseudomallei* isolates has not been conducted. Currently, there are only a few studies of the virulence and pathogenesis of environmental isolates. Lee et al. (2010) [64] reported the successful infection of tomato plants by two local soil isolates (Bp 77/96 and Bp 109/96). However, these *B. pseudomallei* isolates did not infect rice plants.

In terms of genome characterization of environmental isolates, the most extensive study was reported by Nandi et al. (2015) [60] involving WGS of more than 100 *B. pseudomallei* genomes, including 13 local environmental isolates. These 13 isolates were collected from various sites in Singapore and belonged to only three sequence types (STs), namely ST51, ST300 and ST423 (*n* = 7, 1 and 5 respectively), hence implying a much lower diversity than in Thailand, where nine STs could be identified from a single sampling site [65].

## 5. Conclusions

In recent decades, in the clinical setting in Singapore, melioidosis has gained considerable notoriety, with early disease consideration and improved management practices resulting in low mortality rates from the disease. However, it still poses a high threat to animal populations, as early detection and treatment in animals is hindered by variability in clinical course of the disease and the lack of a vaccine for disease prevention. Moving forward, AVA and WRS will be using molecular characterization and typing of the existing isolates to better understand the relationship between the *B. pseudomallei* strains present in the environment and those infecting the different animal populations in Singapore.

Environmental sampling remains a challenge, as isolation of *B. pseudomallei* can be affected by many parameters. Continued efforts in improving protocols and greater support from regulatory authorities for conducting environmental surveillance studies would help identify endemic areas, following which control measures could be applied to reduce disease incidence, particularly in the animal population.

## Figures and Tables

**Table 1:** IHA results on serum samples collected from culture-confirmed melioidosis patients, suspected melioidosis cases presenting to local hospitals and healthy volunteers from 2004 to 2016 [36].

Study Groups	Subject Information/Year of Blood Collection	Number (%) of Subjects with Different Levels of IHA Titer			
		Very High Positive ≥1:512	High Positive 1:128 to 1:256	Low Positive 1:16 to 1:64	Negative ≤1:8
Culture-confirmed melioidosis (*n* = 31)	Acute, relapsed and recovered cases with a mean age of 52.5 years old/2004–2013	17 (54.8%)	12 (38.7%)	2 (6.5%)	0 (0%)
Clinically unconfirmed infection (*n* = 992)	Patients with an active infection/2006	31 (3.1%)	21 (2.1%)	54 (5.4%)	886 (89.4%)
Clinically unconfirmed infection (*n* = 1027)	Patients with an active infection/2016	18 (1.8%)	20 (1.9%)	101 (9.8%)	888 (86.5%)
Healthy volunteers (*n* = 109)	Age range: 18–60 mean age: 33.1 years old/2004–2013	0 (0%)	1 (0.9%)	23 (21.1%)	85 (78.0%)

**Table 2:** Summary of animal melioidosis cases in WRS parks from 1983 to 2017 [50].

	Species	Number	Year of Diagnosis	Organ from Which *B. pseudomallei* Was Isolated						
				Blood	Lung	Liver	Kidney	Spleen	Gonad	Skin
Primates	Gorilla (*Gorilla gorilla*)	5	1983/1992	x	x	x	x			
	Southern pig-tailed macaque (*Macaca nemestrina*)	1	1992		x					
	Chimpanzee (*Pan troglodytes*)	2	1985/1990		x		x			
	Müller’s Bornean gibbon (*Hylobates muelleri*)	2	1989/1992		x			x		
	Mandrill (*Mandrillus sphinx*)	1	1990					x		
	Golden lion tamarin (*Leontopithecus rosalia*)	2	1995/1996		x	x				
	Siamang (*Symphalangus syndactylus*)	1	2005			x				
	Lesser spot-nosed guenon (*Cercopithecus petaurista*)	1	1996		x					
	Debrazza’s monkey (*Cercopithecus neglectus*)	1	1998		x					
	Douc langur (*Simia nemaeus*)	1	1992		x					
Herbivores	Eastern grey kangaroo (*Macropus giganteus*)	2	1986/1989		x		x			
	Indochinese hog deer (*Hyelaphus annamiticus*)	1	2013		x					
	Camel (*Camelus dromedaries*)	1	1994		x					
	Llama (*Lama glama*)	1	1994		x					
	Nile hippopotamus (*Hippopotamus amphibious*)	1	1996			x				
	Red lechwe (*Kobus leche*)	2	1998/2007			x		x		
	Indian sambar (*Rusa unicolor*)	1	2003			x				
	Chinese goral (*Naemorhedus griseus*)	1	2008							x
Carnivores	Cape hunting dog (*Lycaon pictus pictus*)	1	1990	x						
Birds	Southern cassowary (*Casuarius casuarius*)	1	1985					x		
	Southern crowned pigeon (*Goura scheepmakeri*)	1	1987						x	
	Moustached parakeet (*Psittacula alexandri*)	1	1998			x				
	Palm cockatoo (*Proboscigera terrimus*)	2	1991/1996			x				
	Salmon-crested cockatoo (*Cacatua moluccensis*)	2	1998/2014			x				
	Humboldt penguin (*Spheniscus humboldti*)	1	1987			x				
	Bird of paradise (exact species unknown)	1	1986			x				

x: organ of animal species where *B. pseudomallei* was isolated.

**Table 3:** Literature review of environmental surveillance for *B. pseudomallei* in Singapore.

Type of Environmental Sample	Terrain/Location	Isolation Protocol	No. Collected	No. Positive	Percentage (%)	Interesting Correlations *	Reference
Surface water	Forest around buildings, roadside drains, sports fields	Hamster inoculation method [55]; colony identification on MacConkey agar.	21 44 43 28	1 0 2 5	4.8 0.0 4.6 18.0	- - Rainfall Rainfall; low lying field	[53]
	Different localities where melioidosis patients had sustained injuries or had direct contact prior to their onset of illness	TSB with crystal violet (5 mg/L) and colistin (20 mg/L); colony identification on Ashdown agar.	46	0	0.0	-	[16]
Water	Moat within animal enclosure in Singapore Zoological Gardens (WRS)	Hamster inoculation method [55]; colony identification on MacConkey’s agar.	Unknown	3	N.D.	4 gorillas succumbed to melioidosis; both animal and water isolates belonged to same RE II type.	[54]
Soil	Island off Singapore Residential compound	Hamster inoculation method [55]; colony identification on MacConkey agar.	Unknown 4	2 1	N.D. 25.0	2 patients staying on the island died of melioidosis (see Table 5). Pet German shepherd succumbed to melioidosis; both animal and soil isolates belonged to same RE II type.	[54]
	Different localities where melioidosis patients had sustained injuries or had direct contact prior to their onset of illness	TSB with crystal violet (5 mg/L) and colistin (20 mg/L) enrichment; colony identification on Ashdown agar.	395	7	1.8	Genotype (by REA-PFGE) of Bp isolated from 4 samples in 1 locality similar to that isolated from elbow abscess of adult who had sustained injury at same locality.	[16]

* Correlations observed or hypothesized by the authors.

**Table 4:** Environmental surveillance of *B. pseudomallei* in Singapore between 2000 and 2013 [56].

Sample Source	Terrain/Location	Year	No. Collected	No. Positive	Percentage Positive (%)	Type of Study	Interesting Correlations *
Soil	Fields, island	2000/01	98	0	0	Repeated Sampling at sites reported by Yap et al. [54]	Refer to Table 5
	Plantations, island	2000/01	62	3	3.1	Repeated Sampling at sites reported by Yap et al. [54]	
	Reclaimed land, island	2000/01	28	0	0	Repeated Sampling at sites reported by Yap et al. [54]	
	Animal enclosure 1	2001	18	0	0	Collaboration with SZG.	
	Animal enclosure 2	2001	13	0	0	Collaboration with SZG.	
	Animal enclosure 3	2001	8	0	0	Collaboration with Dr. Paul A. Tambyah, NUH	
	Animal enclosure 4	2001	4	0	0	Collaboration with Dr. Paul A. Tambyah, NUH	
	Park A	2005	15	0	0	Environmental Surveillance at 9 locations	
	Park B	2005	5	0	0	Environmental Surveillance at 9 locations	
	Park C	2005	90	3	3.3	Environmental Surveillance at 9 locations	
	Park D	2005	100	1	1.0	Environmental Surveillance at 9 locations	
	Nature Reserve A	2005	79	0	0	Environmental Surveillance at 9 locations	
	Nature Reserve B	2005	30	0	0	Environmental Surveillance at 9 locations	
	Nature Reserve C	2005	90	0	0	Environmental Surveillance at 9 locations	
	Disturbed soil area A	2005	259	0	0	Environmental Surveillance at 9 locations	
	Disturbed soil area B	2005	63	0	0	Environmental Surveillance at 9 locations	
	Forested hill, southern island	2013	55	1	1.8	Environmental Surveillance project in 2013	
Water	Water from streams/puddles, island	2000/01	16	2	12.5	Repeated sampling at sites reported by Yap et al. [54]	Refer to Table 5
	Moat within animal enclosure 2	2001	3	0	0	Collaboration with SZG.	
	Water behind animal enclosure 3	2001	1	0	0	Collaboration with Dr Paul A. Tambyah, NUH	
	Run-off from forested hill, southern island	2013	1	0	0	Environmental surveillance project in 2013	
Rainwater	Forested hill, southern island	2013	9	0	0	Environmental Surveillance project in 2013	

SZG-Singapore Zoological Gardens; * Correlations observed or hypothesized by the authors.

**Table 5:** Correlation of clinical and environmental *B. pseudomallei* isolates from an island off Singapore [61].

Isolate	Source	Isolated by	Accessory Genome Clade [47]	Sequence Type (MLST) [60]	Genomic Clade (WGS) [60]
22	Human, clinical	Yap et al. (1995) [54]	Environmental clade	ST423	N.D.
DB	Soil, environmental	Yap et al. (1995) [54]	Animal clade	ST51	A
DC	Soil, environmental	Yap et al. (1995) [54]	Animal clade	ST51	A
15-10	Soil, environmental	DSO 2000/01	Environmental clade	ST423	B
15-40	Soil, environmental	DSO 2000/01	Environmental clade	ST423	B
12-40	Soil, environmental	DSO 2000/01	Environmental clade	ST423	B
SW1	Water, environmental	DSO 2000/01	Environmental clade	ST423	B
SW9	Water, environmental	DSO 2000/01	Environmental clade	ST423	B

## References

[1] Dance D.A. (1991). Melioidosis: The tip of the iceberg?. Clin. Microbiol. Rev..

[2] Thomas A.D., Spinks G.A., D’Arcy T.L., Norton J.H., Trueman K.F. (1988). Evaluation of four serological tests for the diagnosis of caprine melioidosis. Aust. Vet. J..

[3] Cheng A.C., Currie B.J. (2005). Melioidosis: Epidemiology, pathophysiology, and management. Clin. Microbiol. Rev..

[4] White N.J. (2003). Melioidosis. Lancet.

[5] Chan H.P., Yip H.S. (2015). Mediastinal lymphadenopathy: Melioidosis mimicking tuberculosis. Trop. Med. Health.

[6] Wijekoon S., Prasath T., Corea E.M., Elwitigala J.P. (2014). Melioidosis presenting as lymphadenitis: A case report. BMC Res. Notes.

[7] Choy J.L., Mayo M., Janmaat A., Currie B.J. (2000). Animal melioidosis in Australia. Acta. Trop..

[8] Dance D.A. (2000). Melioidosis as an emerging global problem. Acta. Trop..

[9] Elschner M.C., Hnizdo J., Stamm I., El-Adawy H., Mertens K., Melzer F. (2014). Isolation of the highly pathogenic and zoonotic agent *Burkholderia pseudomallei* from a pet green iguana in Prague, Czech Republic. BMC Vet. Res..

[10] Moore R.A., Tuanyok A., Woods D.E. (2008). Survival of *Burkholderia pseudomallei* in water. BMC Res. Notes.

[11] Pumpuang A., Chantratita N., Wikraiphat C., Saiprom N., Day N.P., Peacock S.J., Wuthiekanun V. (2011). Survival of *Burkholderia pseudomallei* in distilled water for 16 years. Trans. R. Soc. Trop. Med. Hyg..

[12] Stanton A.T., Fletcher W. (1932). Melioidosis. Studies from the institute of medical research, Federated Malay States. Bull.

[13] Limmathurotsakul D., Golding N., Dance D.A., Messina J.P., Pigott D.M., Moyes C.L., Rolim D.B., Bertherat E., Day N.P., Peacock S.J. (2016). Predicted global distribution of *Burkholderia pseudomallei* and burden of melioidosis. Nat. Microbiol..

[14] Tan A.L., Ang B.S., Ong Y.Y. (1990). Melioidosis: Epidemiology and antibiogram of cases in Singapore. Singap. Med. J..

[15] Lim M.K., Tan E.H., Soh C.S., Chang T.L. (1997). *Burkholderia pseudomallei* infection in the Singapore Armed Forces from 1987 to 1994 - an epidemiological review. Ann. Acad. Med. Singap..

[16] Heng B.H., Goh K.T., Yap E.H., Loh H., Yeo M. (1998). Epidemiological surveillance of melioidosis in Singapore. Ann. Acad Med. Singap..

[17] Lo T.J., Ang L.W., James L., Goh K.T. (2009). Melioidosis in a tropical city state, Singapore. Emerg. Infect. Dis..

[18] Pang L., Harris P.N.A., Seiler R.L., Ooi P.L., Cutter J., Goh K.T., Cook A.R., Fisher D., Chai L.Y.A. (2018). Melioidosis, Singapore, 2003–2014. Emerg. Infect. Dis..

[19] Liu Y., Loh J.P., Aw L.T., Yap E.P., Lee M.A., Ooi E.E. (2006). Rapid molecular typing of *Burkholderia pseudomallei*, isolated in an outbreak of melioidosis in Singapore in 2004, based on variable-number tandem repeats. Trans. R. Soc. Trop. Med. Hyg..

[20] Yip S.K., Ang B.S., Tan J. (2001). Clinics in diagnostic imaging (57). Singap. Med. J..

[21] Tan A.P., Pui M.H., Tan L.K. (1995). Imaging patterns in melioidosis. Australas. Radiol..

[22] Ng W.M., Kwan M.K., Merican A.M. (2006). Melioidotic osteomyelitis treated with antibiotic-calcium hydroxyapatite composite: Case report with four-year follow-up. Singap. Med. J..

[23] Amadasi S., Dal Zoppo S., Bonomini A., Bussi A., Pedroni P., Balestrieri G., Signorini L., Castelli F. (2015). A case of melioidosis probably acquired by inhalation of dusts during a helicopter flight in a healthy traveler returning from Singapore. J. Travel. Med..

[24] Li P.H., Chau C.H., Wong P.C. (2015). Melioidosis mycotic aneurysm: An uncommon complication of an uncommon disease. Respir. Med. Case Rep..

[25] Rao J., Kaushal A.S., Hoong C.K. (2009). Abdominal aortic pseudoaneurysm secondary to melioidosis. Asian J. Surg..

[26] Chlebicki M.P., Kurup A., Sin Y.K. (2008). *Burkholderia pseudomallei* meningitis following inadequate treatment of melioidotic mycotic aneurysm. Singap. Med. J..

[27] Lath R., Rajshekhar V., George V. (1998). Brain abscess as the presenting feature of melioidosis. Br. J. Neurosurg..

[28] Chadwick D.R., Ang B., Sitoh Y.Y., Lee C.C. (2002). Cerebral melioidosis in Singapore: A review of five cases. Trans. R. Soc. Trop. Med. Hyg..

[29] Limmathurotsakul D., Peacock S.J. (2011). Melioidosis: A clinical overview. Br. Med. Bull..

[30] Chong W.S. (2013). Dermatology in the military field: What physicians should know?. World J. Clin. Cases.

[31] Foong Y.W., Tan N.W., Chong C.Y., Thoon K.C., Tee N.W., Koh M.J. (2015). Melioidosis in children: A retrospective study. Int. J. Dermatol..

[32] Meumann E.M., Novak R.T., Gal D., Kaestli M.E., Mayo M., Hanson J.P., Spencer E., Glass M.B., Gee J.E., Wilkins P.P. (2006). Clinical evaluation of a type III secretion system real-time PCR assay for diagnosing melioidosis. J. Clin. Microbiol..

[33] Chantratita N., Wuthiekanun V., Limmathurotsakul D., Thanwisai A., Chantratita W., Day N.P., Peacock S.J. (2007). Prospective clinical evaluation of the accuracy of 16s rRNA real-time PCR assay for the diagnosis of melioidosis. Am. J. Trop. Med. Hyg..

[34] Wuthiekanun V., Chierakul W., Langa S., Chaowagul W., Panpitpat C., Saipan P., Thoujaikong T., Day N.P., Peacock S.J. (2006). Development of antibodies to *Burkholderia pseudomallei* during childhood in melioidosis-endemic northeast Thailand. Am. J. Trop. Med. Hyg..

[35] Yap E.H., Chan Y.C., Ti T.Y., Thong T.W., Tan A.L., Yeo M., Ho L.C., Singh M. (1991). Serodiagnosis of melioidosis in Singapore by the indirect haemagglutination test. Singap. Med. J..

[36] Liu Y., Sim S.H., Wang D. (2018). IHA results on serum samples collected from culture-confirmed melioidosis patients, suspected melioidosis cases presenting to local hospitals and healthy volunteers from 2004 to 2016.

[37] Wiersinga W.J., Currie B.J., Peacock S.J. (2012). Melioidosis. N. Engl. J. Med..

[38] Chierakul W., Anunnatsiri S., Short J.M., Maharjan B., Mootsikapun P., Simpson A.J., Limmathurotsakul D., Cheng A.C., Stepniewska K., Newton P.N. (2005). Two randomized controlled trials of ceftazidime alone versus ceftazidime in combination with trimethoprim-sulfamethoxazole for the treatment of severe melioidosis. Clin. Infect. Dis..

[39] Kung C.T., Lee C.H., Li C.J., Lu H.I., Ko S.F., Liu J.W. (2010). Development of ceftazidime resistance in *Burkhoderia pseudomallei* in a patient experiencing melioidosis with mediastinal lymphadenitis. Ann. Acad. Med. Singap..

[40] Fisher D. (2008). Division of infectious diseases, Department of Medicine, National University Health System, Singapore.

[41] Suputtamongkol Y., Chaowagul W., Chetchotisakd P., Lertpatanasuwun N., Intaranongpai S., Ruchutrakool T., Budhsarawong D., Mootsikapun P., Wuthiekanun V., Teerawatasook N. (1999). Risk factors for melioidosis and bacteremic melioidosis. Clin. Infect. Dis..

[42] Liu X., Foo G., Lim W.P., Ravikumar S., Sim S.H., Win M.S., Goh J.G., Lim J.H., Ng Y.H., Fisher D. (2014). Sulphonylurea usage in melioidosis is associated with severe disease and suppressed immune response. PLoS Negl. Trop. Dis..

[43] (2017). Annual Report 2016/17: Handled with Care.

[44] (2016). Pet Care in Singapore, Industry Overview.

[45] Sprague L.D., Neubauer H. (2004). Melioidosis in animals: A review on epizootiology, diagnosis and clinical presentation. J. Vet. Med. B. Infect. Dis. Vet. Public Health.

[46] Tonpitak W., Sornklien C., Chawanit M., Pavasutthipaisit S., Wuthiekanun V., Hantrakun V., Amornchai P., Thaipadungpanit J., Day N.P., Yingst S. (2014). Fatal melioidosis in goats in Bangkok, Thailand. Am. J. Trop. Med. Hyg..

[47] Sim S.H., Yu Y., Lin C.H., Karuturi R.K., Wuthiekanun V., Tuanyok A., Chua H.H., Ong C., Paramalingam S.S., Tan G. (2008). The core and accessory genomes of *Burkholderia pseudomallei*: Implications for human melioidosis. PLoS Pathog..

[48] Currie B.J., Fisher D.A., Howard D.M., Burrow J.N., Selvanayagam S., Snelling P.L., Anstey N.M., Mayo M.J. (2000). The epidemiology of melioidosis in Australia and Papua New Guinea. Acta Trop..

[49] Ketterer P.J., Webster W.R., Shield J., Arthur R.J., Blackall P.J., Thomas A.D. (1986). Melioidosis in intensive piggeries in south eastern Queensland. Aust. Vet. J..

[50] Xie S. (2018). Conservation, Research and Veterinary Services, Wildlife Reserves Singapore, Singapore.

[51] Walsh A.L., Wuthiekanun V., Smith M.D., Suputtamongkol Y., White N.J. (1995). Selective broths for the isolation of *Pseudomonas pseudomallei* from clinical samples. Trans. R. Soc. Trop. Med. Hyg..

[52] Supaprom C., Wang D., Leelayuwat C., Thaewpia W., Susaengrat W., Koh V., Ooi E.E., Lertmemongkolchai G., Liu Y. (2007). Development of real-time PCR assays and evaluation of their potential use for rapid detection of *Burkholderia pseudomallei* in clinical blood specimens. J. Clin. Microbiol..

[53] Thin R.N., Groves M., Rapmund G., Mariappan M. (1971). *Pseudomonas pseudomallei* in the surface water of Singapore. Singap. Med. J..

[54] Yap E.H., Thong T.W., Tan A.L., Yeo M., Tan H.C., Loh H., Teo T.P., Thong K.T., Singh M., Chan Y.C. (1995). Comparison of *Pseudomonas pseudomallei* from humans, animals, soil and water by restriction endonuclease analysis. Singap. Med. J..

[55] Strauss J.M., Groves M.G., Mariappan M., Ellison D.W. (1969). Melioidosis in Malaysia. Ii. Distribution of *Pseudomonas pseudomallei* in soil and surface water. Am. J. Trop. Med. Hyg..

[56] Ong C.E.L., Koh V.W.H., Tan Y.K., Wong M.S.Y., Chew J.S.W. (2018). Environmental surveillance of B. pseudomallei in Singapore between 2000 and 2013.

[57] Chantratita N., Wuthiekanun V., Boonbumrung K., Tiyawisutsri R., Vesaratchavest M., Limmathurotsakul D., Chierakul W., Wongratanacheewin S., Pukritiyakamee S., White N.J. (2007). Biological relevance of colony morphology and phenotypic switching by *Burkholderia pseudomallei*. J. Bacteriol..

[58] Limmathurotsakul D., Wuthiekanun V., Amornchai P., Wongsuwan G., Day N.P., Peacock S.J. (2012). Effectiveness of a simplified method for isolation of *Burkholderia pseudomallei* from soil. Appl. Environ. Microbiol..

[59] Currie B.J., Price E.P., Mayo M., Kaestli M., Theobald V., Harrington I., Harrington G., Sarovich D.S. (2015). Use of whole-genome sequencing to link *Burkholderia pseudomallei* from air sampling to mediastinal melioidosis, Australia. Emerg. Infect. Dis..

[60] Nandi T., Holden M.T., Didelot X., Mehershahi K., Boddey J.A., Beacham I., Peak I., Harting J., Baybayan P., Guo Y. (2015). Burkholderia pseudomallei sequencing identifies genomic clades with distinct recombination, accessory, and epigenetic profiles. Genome Res..

[61] Ong C.E.L. (2018). Correlation of clinical and environmental B. pseudomallei isolates from an island off Singapore.

[62] Sivalingam S.P., Sim S.H., Aw L.T., Ooi E.E. (2006). Antibiotic susceptibility of 50 clinical isolates of *Burkholderia pseudomallei* from Singapore. J. Antimicrob. Chemother..

[63] Tan A.L., Tan M.L. (2008). Melioidosis: Antibiogram of cases in Singapore 1987–2007. Trans. R. Soc. Trop. Med. Hyg..

[64] Lee Y.H., Chen Y., Ouyang X., Gan Y.H. (2010). Identification of tomato plant as a novel host model for *Burkholderia pseudomallei*. BMC Microbiol..

[65] Chantratita N., Wuthiekanun V., Limmathurotsakul D., Vesaratchavest M., Thanwisai A., Amornchai P., Tumapa S., Feil E.J., Day N.P., Peacock S.J. (2008). Genetic diversity and microevolution of *Burkholderia pseudomallei* in the environment. PLoS Negl. Trop. Dis..

